# Heart Rate Dynamics in Patients with Obstructive Sleep Apnea: Heart Rate Variability and Entropy

**DOI:** 10.3390/e21100927

**Published:** 2019-09-24

**Authors:** Lulu Zhang, Mingyu Fu, Fengguo Xu, Fengzhen Hou, Yan Ma

**Affiliations:** 1 Key Laboratory of Biomedical Functional Materials, School of Science, China Pharmaceutical University, Nanjing 210009, China; 2 Key Laboratory of Drug Quality Control and Pharmacovigilance, China Pharmaceutical University, Nanjing 210009, China; 3 Center for Dynamical Biomarkers, Division of Interdisciplinary Medicine and Biotechnology, Beth Israel Deaconess Medical Center, Harvard Medical School, Boston, MA 02215, USA

**Keywords:** electrocardiogram, heart rate dynamics, obstructive sleep apnea, graph theory, entropy

## Abstract

Background: Obstructive sleep apnea (OSA), a highly prevalent sleep disorder, is closely related to cardiovascular disease (CVD). Our previous work demonstrated that Shannon entropy of the degree distribution (E_DD_), obtained from the network domain of heart rate variability (HRV), might be a potential indicator for CVD. Method: To investigate the potential association between OSA and E_DD_, OSA patients and healthy controls (HCs) were identified from a sleep study database. Then E_DD_ was calculated from electrocardiogram (ECG) signals during sleep, followed by cross-sectional comparisons between OSA patients and HCs, and longitudinal comparisons from baseline to follow-up visits. Furthermore, for OSA patients, the association between E_DD_ and OSA severity, measured by apnea-hypopnea index (AHI), was also analyzed. Results: Compared with HCs, OSA patients had significantly increased E_DD_ during sleep. A positive correlation between E_DD_ and the severity of OSA was also observed. Although the value of E_DD_ became larger with aging, it was not OSA-specified. Conclusion: Increased E_DD_ derived from ECG signals during sleep might be a potential dynamic biomarker to identify OSA patients from HCs, which may be used in screening OSA with high risk before polysomnography is considered.

## 1. Introduction

Obstructive sleep apnea (OSA), characterized by cyclic collapse of the upper airway and partial or complete cessation of airflow during sleep, is highly prevalent with prevalence of 34% in men and 17% in women in the general population [1]. OSA patients have increased risks of developing comorbid cardiovascular disease (CVD) and have notorious outcomes related to CVD. It is estimated that 40–60% of patients are OSA with CVD [2]. Therefore, effective and early diagnosis of OSA will contribute substantially not only to the early intervention of OSA but also to the prevention of the complications [3].

Currently, the gold standard for OSA diagnosis is polysomnography (PSG), which is usually conducted in fully equipped and controlled sleep laboratories. In such PSG-based studies, multiple sensors are placed to record simultaneously nasal airflow, respiratory movement, oxygen saturation (SpO2), electroencephalogram (EEG), electro-oculogram (EOG), electromyogram (EMG), electrocardiogram (ECG) and body position [3]. OSA is diagnosed based on the symptoms and values of apnea–hypopnea index (AHI) of sleep [4]. Although PSG can provide an accurate diagnostic report, the study is expensive and the manual scoring is time-consuming. The need for alternative diagnosis approaches of OSA persists.

As a non-invasive exam, ECG is widely used in clinics [5]. Heart rate variability (HRV) is generally used to describe the variation of intervals between the peaks of successive R-wave in ECG [6] and often called RR intervals. HRV analysis has been recommended as a tool for screening OSA. Increasing number of studies confirmed the fact that healthy heart rate rhythms is complex, non-stationary and nonlinear [7,8,9], thus nonlinear dynamical analysis are more recommended. Compared to healthy subjects, decreased heart rate complexity measured by entropy has been reported as a sensitive indicator for the presence of OSA [10,11,12].

Fractal organization is another important nonlinear feature of healthy cardiac dynamics [13]. Our previous work demonstrated that abnormal activation of the cardiovascular controls with altered fractal organization during sleep might be a potential risk factor for adverse cardiovascular event [14]. Shannon entropy of the degree distribution (E_DD_), obtained from the network domain of HRV, was reported to be an indicator for monitoring the alterations in fractal organization of cardiac dynamics [14,15]. However, to our knowledge, no previous publication has evaluated the performance of this approach to distinguish patterns of HRV from OSA and healthy subjects.

In this study, we identified a group of OSA patients and healthy subjects in an open database. Because differences exist between sleep stages, we separately analyzed heart rate dynamics in rapid eye movement (REM) sleep and Stage 2 sleep, which is the most dominant non-REM sleep stage. We aimed to explore the potential associations between E_DD_ and OSA, by cross-sectional comparisons between groups and longitudinal comparisons from baseline to follow-up visit. Meanwhile, we aimed to investigate whether E_DD_ can be used to detect heart rate alterations in OSA patients.

## 2. Materials and Methods

### 2.1. Data Collection

#### 2.1.1. Participants

Data analyzed in this retrospective study were obtained from the database of Sleep Heart Health Study (SHHS) [16], a multi-center cohort study that was implemented by the National Heart, Lung, and Blood Institute to determine cardiovascular and other consequences of sleep-disordered breathing. Participants were recruited for a baseline PSG in the first exam cycle (SHHS-1, 1995–1998) and a follow-up PSG in the second exam cycle (SHHS-2, 2001–2003).

To determine the association of HRV and OSA, we screened subjects with OSA but otherwise healthy at baseline. We excluded subjects if they: (1) had a value of AHI less than five; (2) drank every day; (3) had a history of diabetes, hypertension, stroke, congestive heart failure, angina, myocardial infarctions, revascularization procedure, myocardial infarction procedure, percutaneous transluminal coronary angioplasties, or coronary artery bypass graft surgeries prior to SHHS-2 exam; (4) took medication within two weeks prior to the exams with known effects on HRV (tricyclic anti-depressants, benzodiazepines, and non-tricyclic antidepressants); or (5) were lost for follow-up or with insufficient ECG data for HRV analysis.

Following the above exclusion criteria, 60 participants with baseline OSA were included in the present study. All of them had both baseline and follow-up PSG recordings. Moreover, healthy controls (HCs), who had a value of AHI less than five at SHHS-1, were selected using the same Exclusion Criteria (2)–(4). As a result, 48 subjects were included in the HC group at SHHS-1, whereas only 18 out of them had follow-up PSGs at SHHS-2. Thus, the longitudinal comparisons for the HCs were conducted in those 18 subjects.

#### 2.1.2. PSG and Sleep Outcomes

Unattended overnight PSG was performed with a portable PS-2 system (Compumedics, Abottsville, Australia). Sensors were placed and equipment was calibrated during an evening home visit by a certified technician, including two channels of EEG (C3/A2 and C4/A1), EOG (right and left), bipolar submental EMG, thoracic and abdominal excursions (inductive plethysmography bands), airflow (by a nasal-oral thermocouple, Protec, Woodinville, WA, USA), finger pulse oximetry (Nonin, Minneapolis, MN, USA), ECG, body position (mercury gauge sensor), and ambient light (on/off, by a light sensor secured to the recording garment). After equipment retrieval, the data were forwarded to a central reading center (Case Western Reserve University, Cleveland, OH) for scoring according to a standard protocol. The polysomnographic methods, scoring protocol, and quality assurance procedures were described in other studies [17,18].

### 2.2. Signal Preprocessing

ECG was sampled at 125 Hz in baseline PSGs, and 250 Hz in follow-up PSGs. For each participant, maximal overlap discrete wavelet transformation was used to extract RR intervals from the ECG signals [19,20]. RR intervals are generally analyzed in a standardized duration of 5 min for short-term HRV analysis. However, most graph theoretic measures, such as E_DD_, are contingent on the number of nodes [21], which was directly determined by data length of the used time series in this study. Considering that heart rate is different among different individuals, we thus used a uniformed data length instead of a uniformed time duration to pick-up all the RR segments. As a data length of 500 has commonly been used in HRV studies [22,23,24], segments with 550 successive RR intervals (approximately 7–9 min duration in sleep) in all sleep stages (REM or Stages 1–4) were extracted. Then, after removing the artifacts or ectopic beats in these segments, a series with 500 data points (also called NN intervals) were used for further HRV analysis. As there were insufficient segments to be analyzed in Stages 1, 3 and 4 sleep for the majority of participants, only HRV in REM and Stage 2 sleep were further analyzed.

### 2.3. Conventional TIME and Frequency Domain HRV Measures

Measures derived from time domain and frequency domain analysis are commonly used in HRV studies [25,26,27]. Thus, in this study, HRV indices, such as mean NN intervals (meanNN), standard deviation of NN intervals (SDNN), percentage of heart period differences larger than 50 ms (pNN50), low frequency (0.04–0.15 Hz) power (LF), high frequency (0.15–0.40 Hz) power (HF), LF in normalized units (LFnorm), HF in normalized units (HFnorm) and total power (TP), were computed.

### 2.4. Nonlinear Measures

#### 2.4.1. Sample Entropy (SampEn)

SampEn was developed as a refinement of the approximate entropy to reduce the bias induced by self-matching. It is independent of the length of recording and exhibits relative consistency under various circumstances [28]. For a time series with *N* data points, denoted as {x(n)|n=1,2,…,N}, to compute the SampEn, vectors are first constructed in the delayed *m*-dimensional space as:

(1) $$X_{m}\left(i\right)=\left\{x\left(i\right),x\left(i+1\right),\ldots ,x\left(i+m-1\right)\right\}\left(i=1,2,\ldots ,N-m+1\right).$$

By defining the distance between two vectors $d\left[X_{m}\left(i\right),\;X_{m}\left(j\right)\right]$ as the maximum difference between their corresponding components, for a vector $X_{m}\left(i\right)$, we can calculate the probability of vectors whose distance to $X_{m}\left(i\right)$ is less than a predefined tolerance *r*, as shown in Equation (2),

(2) Aim(r)= {d[Xm(i), Xm(j)]≤r}(N−m)(1≤j≤N−m+1, j≠i).

Then, we can get the average of $A_{i}^{m}\left(r\right)$ when *i* varies from 1 to *N*−*m* + 1 and denoted it as $A^{m}\left(r\right)$, as shown in Equation (3):

(3) Am(r)=∑i=1N−m+1Aim(r)N−m+1

Similarly, as shown in Equation (4), $A^{m+1}\left(r\right)$ can be obtained when the original time series is embedded in (*m* + 1)-dimensional space.

(4) Am+1(r)=∑i=1N−mAim+1(r)N−m

Finally, the SampEn of {x(n)|n=1,2,…,N} can be calculated as:

(5) SampEn(x,m,r)=−ln(Am+1(r)Am(r))

One issue in the calculation of SampEn is to determine the dimension *m* and tolerance *r*. Based on previous studies [29,30], we set *m* to 2 and *r* as 0.15 times the standard deviation of the time series {x(n)|n=1,2,…,N}.

#### 2.4.2. Shannon Entropy of the Degree Distribution (E_DD_)

After converting the original time series into network domain, an important metric of the obtained network, E_DD_, seems promising in capturing the alterations of cardiac dynamics under different physiological and pathological conditions [14,15]. As proposed in the work of Lacasa et al. [31], a time series with *N* data points, denoted as {x(n)|n=1,2,…,N}, can be converted to a network through the visibility graph (VG) algorithm. To carry out the VG algorithm, first, take each data point as a node in the network sequentially. Then, determine connections between all node pairs in the network according to the visibility criteria, which is shown in the conditional expression below:

(6) $$\forall \;k\;\in \left(i,j\right);\;x\left(k\right)<\;x\left(j\right)-\left(j-k\right)\frac{x\left(j\right)-\;x\left(i\right)}{j-i}$$

**Figure 1:**
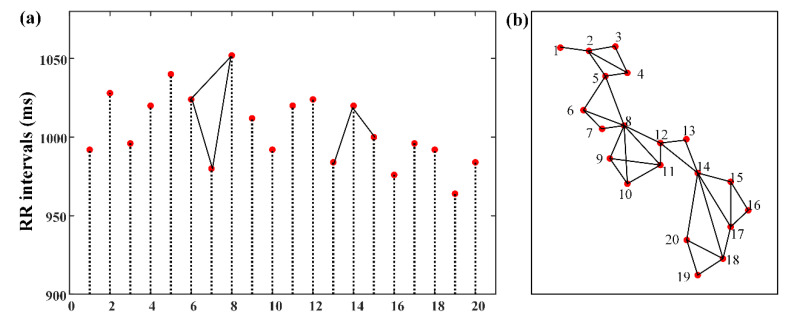
(**a**) Example of an RR-interval time series with 20 data points. Solid lines illustrate the visibility criteria. Stem 6 can be seen from Stems 7 and 8. However, no visibility can be seen between Stems 13 and 15 because sight is blocked by Stem 14. (**b**) The associated graph extracted from time series in (a) using the VG algorithm.

For a pair of nodes, *x*(*i*) and *x*(*j*), they will be connected in the network only when Equation (6) is satisfied. An example for the VG algorithm on a RR-interval time series with 20 data points is illustrated in Figure 1.

Once the network is constructed via the VG algorithm, the degree of each node in the network can be calculated by counting the number of edges incident with the node. E_DD_ can then be obtained as the Shannon entropy of the degree distribution, as shown in Equation (7):

(7) $$E_{DD}=\;-\;\overset{Max\left(k\right)}{\underset{i=0}{\sum }}p\left(k\right)\times \log\;p\left(k\right),$$

where p(k) is defined as the fraction of nodes with degree k in the network and Max(k) as the maximum of k [15].

### 2.5. Statistical Analyses

MATLAB R2012a (Mathworks Inc., Natick, MA) software was used for statistical evaluations. The normality of the data was firstly evaluated by Lilliefors test. For cross-sectional comparisons between the OSA and HC, comparisons of gender were made using the Fisher’s exact test and comparisons of continues variables were assessed by non-parametric test (Mann–Whitney U) if they violate the normality otherwise by t-test. For longitudinal comparisons, sign rank test was used if the data violate the normality, otherwise paired *t*-test was used. A *p*-value < 0.05 was considered as an indicator for significant difference.

## 3. Results

Table 1 summarizes the demographic characteristics of subjects included in this study. At SHHS-1, no significant differences were found in age, gender and BMI. Interestingly, as far as the PSG-based sleep measures were concerned, OSA patients had significantly less sleep in Stage 1 compared with the HCs. At SHHS-2, no significant differences were found in the demographics and sleep measures except for AHI. Longitudinally, for both groups, BMI remained stable in baseline and follow-up. In terms of PSG-based sleep measures, both groups exhibited a prominent deterioration in sleep quality with significantly decreased deep sleep in Stages 3 and 4. Moreover, increased percentage of sleep in Stage 1 and decreased sleep efficiency were found in OSA group (*p* < 0.05). Although there is no significant alteration in AHI for OSA patients, a significant increase of AHI was observed in HCs. Actually, in SHHS-2, 13 out of 18 HCs had an AHI value more than five and less than fifteen, suggesting a development of mild OSA occurred since baseline.

Table 2 and Table 3 summarize the results for HRV analysis. Note that, for each participant, HRV analysis was conducted on all the qualified segments with 500 NN intervals at each sleep stage (REM or Stage 2). Then, the average of each HRV measure during REM or Stage 2 sleep was calculated to provide a single number per subject, which was then subjected to statistical analysis.

**Figure 2:**
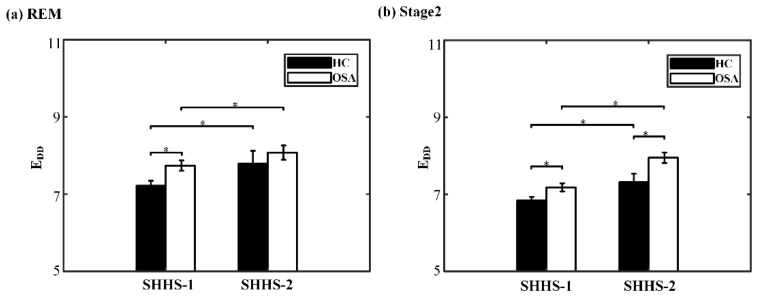
Cross-sectional and longitudinal comparisons of E_DD_ during different sleep stages. (**a**) REM sleep and (**b**) Stage 2 sleep. * indicates *p* < 0.05.

As shown in Table 2, at SHHS-1, compared with HC, significantly increased LFnorm and decreased HFnorm were found in OSA group during Stage 2 sleep. At SHHS-2, OSA patients exhibited a significantly shortened heart rate interval characterized by meanNN. Moreover, as also illustrated in Figure 2, during Stage 2 sleep, significant increases of E_DD_ can be found in OSA related to HC in both SHHS-1 and SHHS-2 visits. During REM sleep, significant difference of E_DD_ between OSA and HC was only observed at SHHS-1.

Regarding the longitudinal alterations of the HRV measures in sleep, it is worth noting that, as shown in Table 3, the majority of HRV measures derived from time domain and frequency domain analysis remained unchanged, even in the OSA group. However, significant increases of E_DD_ were found, regardless of sleep stages or groups (Figure 2). Similarly, significantly decreased SampEn was found in OSA group during both sleep stages, while for the HC, only a significant decline of SampEn was found during REM sleep.

We further investigated the correlation (Pearson’s correlation coefficient) between E_DD_ and AHI for the OSA patients. The results show that a Stage 2-specific positive correlation was found at both baseline (*r* = 0.41, *p* = 0.001) and follow-up (*r* = 0.30, *p* = 0.02) visits.

## 4. Discussion

The present study is an extension of our previous work [14,15], which demonstrated that E_DD_, obtained from HRV signals in network domain, might be a potential risk factor for CVD. As OSA is closely related to CVD, here we calculated E_DD_ and other traditional HRV measures derived from time domain (meanNN, SDNN and pNN50), frequency domain (LF, HF, LFnorm, HFnorm and TP) and nonlinear analysis (SampEn) in OSA patients. Both cross-sectional and longitudinal comparisons were made based on an open database SHHS. Our main findings are threefold. Firstly, compared with HCs, significantly increased E_DD_ during sleep can be found in OSA patients at both baseline and follow-up. Secondly, both OSA and HCs exhibited a significant increase of E_DD_ in the follow-up visit compared with baseline. Thirdly, for OSA patients, a positive correlation between E_DD_ and the severity of disease (characterized by the values of AHI) was found at both baseline and follow-up. As both OSA and aging are risk factors for CVD, our findings might suggest that increased E_DD_ in sleep should be a sensitive indicator associated with the high risk for CVD. No other HRV indices investigated in this study shown coincident and significant alteration with the presence of OSA, aging and deterioration of OSA.

HRV is a physiological variable that is affected by the interaction of the sympathetic and the parasympathetic autonomous systems [32,33]. It is commonly recognized that the HF component of HRV is associated with parasympathetic activity, while the LF component might be an indicator of both sympathetic and parasympathetic activity [26,34,35]. Studies have demonstrated that OSA patients may develop enhanced sympathetic activity due to abnormal adaptability of cardiac autonomic system, leading to a decreased HF and increased LF [12,36]. In accordance with the previous studies, we observed a significant decreased HFnorm and increased LFnorm in the cross-sectional comparisons at baseline. However, in the follow-up visit, the differences in these HRV metrics between the OSA patients and HCs disappeared while the difference in E_DD_ maintained from baseline to follow-up during Stage 2 sleep. As shown in Table 1, there is a significant increase of AHI for the HCs from SHHS-1 to SHHS-2, suggesting that the majority of the HCs (13 out of 18) had actually developed to mild OSA. Such an alteration might contribute to the insensitive of the investigated frequency metrics of HRV to differentiate OSA and HCs in SHHS-2.

Actually, heart rate is influenced by numerous interacting factors, including not only the sympathetic and parasympathetic activity, but also the physical activity, hormonal and temperature variations, digestion, circadian rhythms and so on, leading to exceedingly complex variations in healthy heart rate dynamics [37]. In recent years, along with system complexity becoming an established theory in health science, decreased complexity has been recognized as a common indicator of pathological conditions or aging [38,39]. Entropy-based measures have been widely used for the evaluation of complexity [10,11,40]. Amongst them, SampEn is frequently adopted in the analysis of clinical time series, such as HRV, ECG and EEG signals [28,41,42]. Numerous studies have demonstrated that biological aging is characterized by a loss of physiologic complexity in the dynamics of the cardiovascular [2,43]. Consistently, in our study, decreased SampEn was found in the follow-up visit compared with those at the baseline, for both OSA patients and HCs. However, in our observation, SampEn was not sensitive for capturing the difference between OSA and HC in sleep.

As far as the E_DD_ was concerned, in our previous work, by using HRV data in daytime when subjects were awake, we reported that E_DD_ was significant decreased in pathological individuals (e.g., congestive heart failure, atrial fibrillation and ventricular tachyarrhythmia) compared with healthy controls, revealing a reduction of E_DD_ is possibly a dynamic marker of cardiac disorders [15]. However, in this study, significant increase of E_DD_ in sleep (especially in Stage 2 sleep) was found with the presence of OSA, aging, and the deterioration of OSA. Although future work is required to investigate the mechanism underlying of E_DD_ among different states (sleep or awake), our findings in the present work are consistent with one of our other studies [14]. We previously reported that a larger value of E_DD_ during sleep was observed in older individuals than youth, in males than females, and in overweight participants than those with normal BMI values, suggesting a positive association between increased E_DD_ and the high risks of CVD, such as aging and obesity [14].

Signals from physiological systems (e.g., cardiovascular system) are generally nonlinear and chaotic, coordinated and controlled by a variety of independent factors with complex and nonlinear components [44,45]. The nonlinear analysis of HRV has thus been suggested as a powerful supplement to the conventional time and frequency domain HRV approaches in recent years. This view was supported in the current work as E_DD_ sensitively captured the alterations which may contribute to the presence and deterioration of OSA. Our findings make an addition to existing knowledge by showing that E_DD_ might be a sensitive measure to capture the altered heart rate dynamics in OSA patients. Thus, our study may inspire and encourage future applications of nonlinear HRV analysis in this field.

## 5. Conclusions

The present study investigated the association between OSA and a measure derived from nonlinear analysis, the entropy of the degree distribution E_DD_. Increased E_DD_ in sleep was found in OSA patients compared with HCs, in the aging process of OSA patients, and in the illness deterioration of OSA. However, in the current study, there is a relatively small sample for the healthy controls, especially in SHHS-2. Further prospective studies are encouraged to evaluate OSA in a broader range of population and to apply E_DD_ in the OSA diagnostic and intervention system.

## Figures and Tables

**Table 1:** Baseline characteristics of studied subjects.

	HC			OSA			between-Group (*p*)	
	SHHS-1	SHHS-2	*p*	SHHS-1	SHHS-2	*p*	SHHS-1	SHHS-2
	(*n* = 48)	(*n* = 18)		(*n* = 60)	(*n* = 60)			
*Demographics*								
Age (years)	76[74,78]	82[78,83]	<0.001 *	75[74,77]	80[79,83]	<0.001 *	0.594	0.914
Gender(male/female)	11/37	5/13		14/46	14/46		1	0.758
BMI (kg/m^2^)	26.0 ± 4.1	26.8 ± 4.8	0.589	26.6 ± 4.3	26.2 ± 4.4	0.083	0.488	0.608
*Sleep Measures*								
TST (min)	361 ± 69	359 ± 77	0.265	359 ± 45	355 ± 58	0.834	0.498	0.825
SE (%)	82.4[74.3,88.8]	77.6[67.7,87.4]	0.064	85.8[79.7,90.1]	79[71,84.4]	<0.001 *	0.119	0.688
Stage 1 sleep (%)	4.84[3.31,6.83]	6[5,7]	0.051	3.55[2.3,5.41]	5[3,7]	0.012 *	0.030 *	0.305
Stage 2 sleep (%)	54 ± 10.9	58.7 ± 8	0.136	55.7 ± 11.6	57.2 ± 10.5	0.294	0.438	0.572
Stage 3+4 sleep (%)	20 ± 11.9	13.4 ± 9.4	0.019 *	21.1 ± 12.1	17.7 ± 9.8	0.001 *	0.633	0.111
REM sleep (%)	20.5 ± 5.8	21.4 ± 5.1	0.791	19.7 ± 5.2	20.6 ± 6.5	0.369	0.441	0.63
AHI (event/hour)	2.15[0.82,3.34]	6.92[3.27,10.6]	<0.001 *	13.5[8.8,25.1]	15.5[8.25,24.9]	0.466	<0.001 *	<0.001 *

Note: If the data violate the normality, values are expressed as median [lower quartile, upper quartile], otherwise as mean ± SD. Abbreviations: HC, health control; SHHS, sleep heart health study; BMI, body mass index; TST, total sleep time; SE, sleep efficiency; REM, rapid eye movement; AHI, apnea-hypopnea index. * *p* < 0.05.

**Table 2:** Heart rate variability comparisons between groups during REM and Stage 2 sleep.

Parameter	SHHS-1			SHHS-2		
	HC	OSA	*p*	HC	OSA	*p*
*REM Sleep*						
meanNN	949 ± 112	914 ± 95	0.082	969 ± 120	890 ± 136	0.031 *
SDNN	37.2[28.8,47.8]	43.5[34,52.2]	0.107	40.7 ± 15.6	44.2 ± 14.4	0.381
pNN50	2.43[0.44,6.06]	1.52[0.53,3.9]	0.411	0.77[0.2,6.37]	2.17[0.52,7.65]	0.226
LF	260[148,565]	330[238,622]	0.096	217[117,572]	331[130,513]	0.859
HF	184[60,457]	146[76,239]	0.471	127[69,330]	180[73,514]	0.665
LFnorm	55.2[37.9,74.9]	69.4[51,80.9]	0.069	66.4[44.4,83.4]	68.5[45.7,88.8]	0.665
HFnorm	39.1[26.9,46]	31.9[17.1,41.5]	0.647	42.7[19.6,53.1]	36.9[25.1,47.5]	0.614
TP	1139[712,2349]	1634[938,2273]	0.134	1123[710,2088]	1621[1118,2326]	0.516
SampEn	1.8 ± 0.39	1.67 ± 0.3	0.156	1.55 ± 0.38	1.46 ± 0.34	0.329
E_DD_	7.02[6.68,7.63]	7.54[6.92,8.37]	0.007 *	7.79 ± 1.4	8.08 ± 1.44	0.463
*Stage 2 Sleep*						
meanNN	981 ± 98	943 ± 111	0.066	995 ± 116	902 ± 97	0.001 *
SDNN	33.3[25.7,43.7]	37.7[29.3,48.3]	0.309	33.6[27.6,42.1]	37.1[30.7,47.4]	0.8
pNN50	2.15[0.77,8.29]	2.44[0.96,5.7]	0.497	2[0.5,5.03]	2.68[1.03,6.82]	0.383
LF	349[211,699]	479[274,745]	0.084	347[225,643]	423[279,834]	0.608
HF	233[116,509]	184[126,431]	0.522	171[65.1,330]	290[145,606]	0.128
LFnorm	52.3[40.3,67.1]	58.8[50.9,74.5]	0.008 *	58.8 ± 23.1	60.7 ± 15	0.882
HFnorm	39.4 ± 12.5	33.3 ± 12.5	0.013 *	34.9 ± 16.9	34.9 ± 12.2	0.987
TP	1078[616,1820]	1428[842,2129]	0.198	1008[608,1715]	1496[1114,2488]	0.1
SampEn	1.92 ± 0.29	1.89 ± 0.24	0.583	1.84 ± 0.26	1.72 ± 0.27	0.106
E_DD_	6.84 ± 0.67	7.18 ± 0.81	0.02 *	7.32 ± 0.93	7.95 ± 1.04	0.024 *

Note: If the data violate the normality, values are expressed as median [lower quartile, upper quartile], otherwise as mean ± SD. Abbreviations: HC, health control; meanNN, mean of NN intervals; SDNN, standard deviation of all NN intervals; pNN50, percent of NN intervals more than 50 ms; LF, low frequency (0.04–0.15 Hz) power ms^2^; HF, high frequency (0.15–0.4 Hz) power ms^2^; LFnorm, LF power in normalized units; HFnorm, HF power in normalized units; TP, total power; SampEn, Sample entropy; E_DD_, the Shannon entropy of the degree distribution. * *p* < 0.05.

**Table 3:** Longitudinal comparisons (SHHS-1 vs SHHS-2) during REM and Stage 2 sleep.

Parameter	HC			OSA		
	SHHS-1	SHHS-2	*p*	SHHS-1	SHHS-2	*p*
*REM Sleep*						
meanNN	941 ± 106	969 ± 120	0.121	914 ± 95	890 ± 136	0.096
SDNN	41.5 ± 16.1	40.7 ± 15.6	0.768	43.7 ± 12.4	44.2 ± 14.4	0.809
pNN50	1.35[0.28,6.41]	0.77[0.2,6.37]	0.811	1.52[0.53,3.9]	2.17[0.52,7.65]	0.141
LF	260[191,585]	217[117,572]	0.248	330[238,622]	331[130,513]	0.047 *
HF	133[48,523]	127[69,330]	0.586	146[76,239]	180[73,514]	0.162
LFnorm	59.2[52.4,81.3]	66.4[44.4,83.4]	0.879	69.4[60,80.9]	68.5[45.7,88.8]	0.877
HFnorm	38[30.4,45.8]	42.7[19.6,53.1]	0.913	31.9[17.1,41.5]	36.9[25.1,47.5]	0.033 *
TP	1065[872,2496]	1123[710,2088]	0.372	1634[938,2273]	1621[1118,2326]	0.729
SampEn	1.81 ± 0.31	1.55 ± 0.38	0.001 *	1.67 ± 0.3	1.46 ± 0.34	<0.001 *
E_DD_	7.27 ± 1.08	7.79 ± 1.4	0.02 *	7.54[6.92,8.37]	8[7.11,8.94]	0.017 *
*Stage 2 Sleep*						
meanNN	980 ± 90	995 ± 116	0.543	943 ± 111	902 ± 97	0.006 *
SDNN	29.7[24.9,46]	33.6[27.6,42.1]	0.396	37.7[29.3,48.3]	37.1[30.7,47.4]	0.591
pNN50	2.49[0.5,8.58]	2[0.5,5.03]	0.372	2.44[0.96,5.7]	2.68[1.03,6.82]	0.627
LF	305[207,608]	347[225,643]	0.267	479[274,745]	423[279,834]	0.489
HF	190[95,544]	171[65,330]	0.372	184[126,431]	290[145,606]	0.227
LFnorm	54.2 ± 17.7	58.8 ± 23.1	0.14	58.8[50.9,74.5]	61.8[50.4,69.8]	0.752
HFnorm	39.7 ± 13.8	34.9 ± 16.9	0.044 *	33.3 ± 12.5	34.9 ± 12.2	0.387
TP	744[615,1959]	1008[608,1715]	0.446	1428[842,2129]	1496[1114,2488]	0.691
SampEn	1.95 ± 0.25	1.84 ± 0.26	0.177	1.89 ± 0.24	1.72 ± 0.27	<0.001 *
E_DD_	6.75 ± 0.7	7.32 ± 0.93	0.004 *	7.18 ± 0.81	7.95 ± 1.04	<0.001 *

Note: If the data violate the normality, values are expressed as median [lower quartile, upper quartile], otherwise as mean ± SD. Abbreviations: HC, health control; meanNN, mean of NN intervals; SDNN, standard deviation of all NN intervals; pNN50, percent of NN intervals more than 50 ms; LF, low frequency (0.04–0.15 Hz) power ms^2^; HF, high frequency (0.15–0.4 Hz) power ms^2^; LFnorm, LF power in normalized units; HFnorm, HF power in normalized units; TP, total power; SampEn, Sample entropy; E_DD_, the Shannon entropy of the degree distribution. * *p* < 0.05.
